# Impact of Practical Online Lessons on Chinese Medical Students’ Perception of Radiation Oncology

**DOI:** 10.1007/s13187-023-02361-1

**Published:** 2023-09-06

**Authors:** Ziye Zheng, Yuxuan Wang, Fuquan Zhang, Jiawei Zhu, Jing Shen, Qingyu Meng, Bei Wang, Bing Zhou, Wei Tian, Lihua Yu, Junfang Yan

**Affiliations:** 1 Department of Radiation Oncology, Peking Union Medical College Hospital, Chinese Academy of Medical Sciences & Peking Union Medical College, No.1 Shuaifuyuan Wangfujing, Dongcheng District, Beijing, 100730 China; 2 Peking Union Medical College, M.D. Program, No. 9 Dongdansantiao, Beijing, 100730 China; 3 Department of Radiation Oncology, State Key Laboratory of Complex Severe and Rare Diseases, Peking Union Medical College Hospital, Chinese Academy of Medical Science and Peking Union Medical College, Beijing, China

**Keywords:** Radiation oncology, Radiotherapy, Medical students, MOOC

## Abstract

Radiotherapy is an essential component of oncology treatment. It is imperative that clinicians and medical students have a fundamental understanding of radiotherapy. However, radiation oncology education is deficient worldwide. This study introduced an hour-long online Massive Open Online Course (MOOC) as a supplement to the basic curriculum for 8-year medical students at Peking Union Medical College and Tsinghua University in China. The students’ personal opinions and comprehension of radiation oncology therapy were assessed through pre- and post-test questionnaires before and after the MOOC study. The results indicated that the percentage of students interested in radiotherapy increased, and their knowledge of radiotherapy significantly improved after the online MOOC study, suggesting that short-term MOOC study may stimulate students’ interest in learning and improving their knowledge of radiation therapy. The study suggests that the combination of online and offline teaching may be a feasible way to develop radiation oncology education in the future.

## Introduction

Cancer ranks as a leading cause of mortality globally, with an estimated 19.3 million new cases and 10.0 million deaths in 2020 [1]. Almost 70% cancer patients receive radiotherapy as a component of treatment. With the rapid advancement of radiotherapy techniques, such as intensity-modulated and image-guided radiation therapy (IMRT and IGRT), its use has become even more widespread. Approximately one in three people will develop cancer during their lifetime, and four out of ten cancer patients will receive radiotherapy as part of their treatment [2].

Given the prevalence of cancer and the widespread use of radiotherapy in its treatment, it is essential for physicians and medical students to have a basic understanding of oncology and radiotherapy. Nonetheless, there is a significant unmet need for oncology education worldwide, particularly in the field of radiation oncology. Surveys conducted in the USA, UK, Spain, Canada, and other countries have indicated that radiation oncology was underrepresented in undergraduate curricula and poorly understood by students [2–5]. The main reasons they gave for the unsatisfied teaching effect including lack of attention, inadequate resource investment, and limited means. Not only are there few class hours dedicated to oncology education — representing only 1/5 to 4/5 of the oncology learning hours for medical students in American and British medical schools, with practical courses being even scarcer [6, 7], but also there is a lack of corresponding teaching research [8, 9].

In China, radiation oncology education appears to be particularly neglected. This highly specialized field requires a strong foundation in clinical oncology, radiation physics, and radiation biology, which were difficult to get access from other fields. Besides, the rapid development of advanced radiotherapy equipment and technologies necessitates continuous learning. However, due to the demanding course load and academic pressure faced by medical students in China, particularly those in 8-year programs, it can be challenging for them to take on such a complex and specialized course that may not align with their future career goals. The COVID-19 crisis has further complicated face-to-face teaching and highlighted the need for digital teaching formats. To address these challenges, in this study, we introduced a Massive Open Online Course (MOOC) teaching format, which allows for shorter learning times and the ability to learn in fragmented time periods, to take advantage of the convenience of online education.

MOOC is a large-scale online virtual classroom that connects instructors located around the world with learners located in various parts of the world through teaching and learning. The concept of MOOC was first proposed as early as 2008 [10]. In 2012, with the rise of three major course providers, Coursera, Udacity, and edX, top American universities began establishing online learning platforms to offer free courses on the internet, providing more students with the possibility of systematic learning. Compared to other online learning methods, MOOCs offer free, high-quality resources that can be shared without geographical restrictions, promoting comprehensive and lifelong learning for students. MOOC courses are carefully designed and include self-practice, exams, and discussion forums to facilitate student self-assessment and further learning and review. In addition, MOOC course certificates are highly recognized, allowing for more independent learning [11]. Previously, MOOCs have been extensively used in medical education and have achieved certain success [12–14], but there have been no reports of MOOCs being used in radiation oncology education. This study attempts to incorporate MOOCs as a supplement to offline courses and observe their teaching effectiveness.

## Methods

This study was conducted from January 2021 to July 2022 in Peking Union Medical College Hospital (PUMCH). Eight-year program medical students from PUMCH and Tsinghua University were enrolled in this research. We administered a pre-test and post-test questionnaire, with the timing of the pre-test commencing at the conclusion of the clinical oncology course, including a 3-credit hour theory course in radiation oncology. This was during the fifth year for PUMC students and the sixth year for Tsinghua University students. Ethical approval was obtained from the Institutional Review Board of the Peking Union Medical College Hospital (No. JS-2373) and each participant provided written informed consent.

The majority of the pre-test and post-test questionnaires were identical and comprised two categories of questions. The first category pertained to students’ personal opinions and understanding of radiation oncology and included seven questions on enjoyment, attitude, authenticity, learning efficacy, and intention to use. For the most part, students were asked to select one answer on a 5-point Likert scale ranging from “strongly agree” to “strongly disagree.” The second category consisted of five questions testing radiotherapy knowledge, including three single-answer and two multiple-answer multiple-choice questions (Table 1). The answers to these questions could not be directly obtained from previous theoretical courses, demonstration courses, or the internet. Students need to comprehend the course content in order to answer them correctly. Questions were designed based on the objectives of the course.

**Table 1:** The five questions testing students’ radiotherapy knowledge

	Pre-test	Post-test
Question 1: What diseases do you think could be treated by radiotherapy alone? (Multiple choice: ABD)		
Relieve pain and symptoms of malignant tumor patients	72 (97.3%)	72 (97.3%)
Completely cure of malignant tumors	64 (86.49%)	68 (91.89%)
Completely cure of benign tumors	61 (82.43%)	62 (83.78%)
Treat certain benign diseases and relieve symptoms	63 (85.14%)	64 (86.49%)
Question 2: What kind of work do you think a radiation oncologist should be primarily engage in? (Single choice: B)		
Diagnosis and staging of tumors by imaging methods such as CT/MRI	6 (8.11%)	1 (1.35%)
Contouring the target area and normal tissue, and restricting the dose	46 (62.16%)	62 (83.78%)
Placing the treatment catheter into the patient’s body or blood vessel under the guidance of X-ray for drug injection or embolization to treat the tumor	6 (8.11%)	0
Diagnosis of tumors with drugs containing radioactive elements	0	0
Using computer software to design the ray incident angle to achieve the purpose of tumor treatment and protection of normal tissue	12 (16.22%)	5 (6.76%)
Determine the patient’s position on the radiation therapy device (accelerator) and maneuver the device to treat as planned	4 (5.41%)	6 (8.11%)
Question 3: Which of the following items do you think can best describe IGRT? (Single choice: C)		
Quickly adjust the position of the jaw and multi-leaf grating according to the irregular tumor shape, so as to be more lined with the tumor shape	4 (5.41%)	0
According to the relationship between the irregular tumor shape and surrounding normal organs, quickly rotate the gantry to adjust the position of the lead gate and multi-leaf grating, so as to make the Irradiation area closer to the tumor shape and better protect the normal organs	20 (27.3%)	19 (25.68%)
Through real-time image acquisition to calibrate with planned images, making the irradiation position change rapidly with the changes in tumor and normal organ positions	35 (47.3%)	43 (58.11%)
Through real-time image acquisition, matching and calibration with the planned image to ensure that the pre-designed rapid adjusted jaw gate and multi-leaf grating positions can be implemented more accurately	15 (20.27%)	12 (16.22%)
Question 4: Which tumor can be cured by radiotherapy alone (without other treatments)? (Multiple choice: ACD)		
Early-stage cervical cancer	61 (82.43%)	69 (93.24%)
Breast cancer	40 (54.05%)	24 (32.43%)
Early-stage throat carcinoma	53 (71.62%)	56 (75.68%)
Early-stage nasopharyngeal carcinoma	61 (82.43%)	69 (93.24%)
Rectum cancer	23 (31.08%)	20 (27.03%)
Question 5: Which of the following is not a common toxic effect of pelvic radiotherapy? (Single choice: B)		
Diarrhea	22 (29.73%)	5 (6.76%)
Constipation	23 (31.08%)	35 (47.3%)
Frequency, urgency, and dysuria	11 (14.86%)	1 (1.35%)
Redness and darkening of the skin	18 (24.32%)	33 (44.59%)

The MOOCs consisted of two parts of practical online lessons, totaling 1 h. The first part briefly introduced clinical cases of radiotherapy application in dominant diseases of the head, neck, chest, abdomen, pelvic cavity, and four kinds of benign diseases. It was interspersed with the introduction to the basic concepts and principles of different radiotherapy techniques, such as intensity modulated radiotherapy, three-dimensional conformal radiotherapy, electron beam radiotherapy, aimed to enable students to master the indications of radiotherapy in a short time. The second part introduced the key steps in the radiation therapy process for a patient with cervical cancer from initial outpatient consultation to follow-up examination, introduced the knowledge about radiotherapy positioning, target area contouring and planning design, alignment, common side effects during radiotherapy treatment, follow-up precautions, and so on. By using short videos as a substitute for offline practice, we tried to provide students with an intuitive understanding of how to implement radiotherapy.

Data were compared using candidate numbers and numerical values ranging from 1 (strongly disagree) to 5 (strongly agree) were assigned. Descriptive statistics were exported into Excel to calculate, and statistical analysis was completed on SPSS for Windows (version 23.0; IBM Corp., Armonk, NY, USA). Categorical variables were described by frequencies and compared by the chi-square test. The total score of knowledge test before and after the practical MOOCs was analyzed by matched samples *t*-test. Statistical significance was set at a two-sided *P*-value < 0.05.

## Results

### Participant Characteristics

Of the 91 preclinical medical students who completed the clinical oncology course, 75 participated in the pre-test. One student who withdrew from the MOOCs was excluded. The response rate was 82.3% (74/91) for all preclinical medical students at PUMCH. Of the participants, 65 were fifth-year students from PUMC and 9 were sixth-year students from Tsinghua University, with response rates of 92.9% and 42.9%, respectively.

### Interest and Exposure to Radiation Oncology

As for the question “Your previous route to study radiation oncology,” didactic lectures (90.54%) were the primary means of delivering radiation oncology knowledge to medical students. Online information (64.86%), academic forum or meetings (37.84%), and research training (9.46%) are less frequently used.

Before the MOOCs learning, 11 students (14.86%) considered radiation oncology to be “very interesting” and 42 students (56.76%) considered it “interesting.” After completing the MOOCs, these numbers increased to 20 students (27.03%) and 45 students (60.81%), respectively. As for the importance of radiotherapy in cancer treatment, 97.30% and 98.65% students chose “important” or “very important” in the pre-text and post-text. Prior to the online practical course, 24 students (32.43%) stated that they might consider a career in radiation oncology. After completing the course, this number increased to 26 students (35.14%), with 2 additional students indicating that they were “very possible” to become a radiation oncologist. In terms of the importance of practical course in the future clinical work, 81.08% students (60/74) thought it was “important” or “very important” before the online practical course, and that increased to 93.24% (69/74) after completing the practical MOOCs (Table 2).

**Table 2:** The questions for student interest. The table presents the number of people and their respective percentages who chose each option in the pre-test and post-test

	Pre-test	Post-test
Question 1. Do you derive enjoyment from radiation oncology?		
Very interesting	11 (14.86%)	20 (27.03%)
Interesting	42 (56.76%)	45 (60.81)
Average	2 (2.7%)	1 (1.35%)
Not interesting	19 (26.68%)	8 (10.8%)
Not at all interesting	0	0
Question 2. How do you perceive the importance of radiation therapy in cancer treatment?		
Very important	50 (67.57%)	52 (70.27%)
Important	22 (29.73%)	21 (28.38%)
Average	2 (2.7%)	1 (1.35%)
Not important	0	0
Not at all important	0	0
Question 3. Do you have any intention to engage in clinical or research work related to oncology after graduation?		
Yes	30 (40.54%)	32 (43.24%)
No	23 (31.08%)	25 (33.78%)
Not clear	21 (28.38%)	17 (22.97%)
Question 4. What is the likelihood of you pursuing a career in the field of radiation oncology after graduation?		
Very likely	3 (5.88%)	5 (10.2%)
Possible	21 (41.18%)	21 (42.86%)
Average	22 (43.14%)	19 (38.78%)
Unlikely	5 (9.8%)	4 (8.16%)
Absolutely not possible	0	0
Question 5. Adding practical courses after theoretical courses in radiation oncology, how do you perceive their importance for future internships and work?		
Very important	22 (29.73%)	21 (28.38%)
Important	38 (51.35%)	48 (64.86%)
Average	12 (16.22%)	5 (6.76%)
Not important	2 (2.7%)	0
Not at all important	0	0

When asked about the reasons why radiation oncology practical courses were not recommended after theoretical lessons, most students (51.35% and 39.19% in the pre- and post-test, respectively) indicated a lack of time due to the pressure of their studies. However, 44 students (59.46%) suggested adding practical lessons after completing the online courses, compared to 33 students (44.59%) before taking the practical online courses. In the post-test questionnaire, most students identified the advantages of radiation oncology practical online courses as broadening their horizons (74.32%) and consolidating their knowledge of theoretical courses (79.73%). Additionally, 97.30% of students (72/74) “agreed” or “strongly agreed” that practical online courses could accurately and comprehensively present the work content and procedures of radiation oncologists.

### Knowledge in Radiation Oncology

For the entire cohort, improvement was observed on all questions except for one question “what diseases can be treated with radiotherapy.” The accuracy rates before and after completing the online practical courses are shown in Table 3. For the question concerning the work content of radiation oncologists, only 62.2% of students had a correct understanding in the pre-test, improving to 83.8% in the post-test (Table 1). Prior to the course, only 17.6% of students correctly answered the first question about which types of cancer can be cured solely by radiotherapy. Some students thought that breast cancer (54.1%) and rectal cancer (31.1%) could be cured only by radiotherapy. After completing the course, 33.8% of students answered this question correctly. The question regarding common side effects of pelvic radiotherapy showed an improvement from 31.1% to 47.3%.

**Table 3:** The number of people who answered correctly in pre-test and post-test. The percentage in parentheses represents the number of people who answered correctly out of the total number of people. The significance of the difference between the results in pre-test and post-test was evaluated using the chi-square test

	Pre-test	Post-test	*P*-value
Question 1: What diseases do you think could be treated by radiotherapy alone?	5 (6.8%)	4 (5.4%)	1.000
Question 2: What kind of work do you think radiation oncologists primarily engage in?	46 (62.2%)	61 (82.4%)	0.006
Question 3: Which of the following items do you think can best describe IGRT?	35 (47.3%)	42 (56.7%)	0.249
Question 4: Which tumor can be cured by radiotherapy alone (without other treatments)?	13 (17.6%)	25 (33.8%)	0.024
Question 5: Which of the following is not a common toxic effect of pelvic radiotherapy?	23 (31.1%)	34 (45.9%)	0.063

The average total score before participating in the courses was 1.65 (out of a possible 5), while the average score after completing the courses was 2.30. This improvement was statistically significant (*P* < 0.005). No student achieved a perfect score in the pre-test, but two students answered all five questions correctly in the post-test. Only one student scored 4 or higher before participating in the courses, compared to eight students after completing the courses.

## Discussion

As the population continues to age due to development, the incidence of cancer also gradually increases. Radiotherapy plays a crucial role in treating a wide range of cancers, resulting in an increasing demand for radiation oncologists. Furthermore, physicians from other disciplines will have more opportunities to interact with cancer patients, making it essential for physicians to have a basic understanding of radiation oncology. These points highlight the importance of radiation oncology education for both undergraduate and post-graduate students. However, there is a lack of research on the effectiveness of radiation oncology education worldwide. Therefore, this study aimed to assess students’ knowledge and attitudes towards radiation oncology and evaluate the effectiveness of using MOOCs in radiation oncology practical courses.

Several conclusions can be drawn from this study. First, MOOCs significantly increased students’ interest in radiation oncology and encouraged more students to pursue careers as radiation oncologists. Second, MOOCs effectively enhanced students’ knowledge of radiation oncology. At the time of completing the pre-test questionnaires, students had already finished the compulsory lecture-based oncology course. However, the pre-test results suggested that students’ knowledge of radiation oncology was limited, indicating that the lecture-based course alone was insufficient for meeting students’ needs for understanding radiotherapy. In contrast, the post-test results showed that MOOCs improved answer accuracy and average scores and enhanced students’ knowledge of radiation oncology. Therefore, it is necessary to incorporate MOOCs into curriculum planning.

The majority of students agreed that radiation oncology was interesting (87.8%) and important (98.6%), and approximately 75% of students believed that practical courses could help consolidate their knowledge and broaden their horizons. However, only 44.6% of students suggested adding practical lessons before taking practical online courses. The reason for this was that the student development program at PUMC was too tightly scheduled to accommodate new courses or offline practical learning for 8-year program medicine students. With 71 compulsory courses of different subjects already included in the development program, students faced significant curriculum pressure. The use of MOOCs for online practical courses, which offered greater flexibility in terms of time, presented a new way to balance students’ need for gaining more knowledge on radiation oncology with their heavy curriculum load.

Research on the status of radiation oncology education in China is limited. There is a lack of systematic cross-college research to assess the overall state of radiation oncology education in the country. Of the 191 medical schools in China, there are significant differences in curriculum settings, and only a few medical schools offer oncology courses for undergraduates. As a result, most medical undergraduates lack basic knowledge of oncology [15]. Additionally, education resources are considered insufficient, with not enough radiation oncologists available to teach students. During the COVID-19 pandemic, telemedicine was used to address the shortage of medical resources in radiation oncology education in rural areas [16]. Most studies in this area have focused on introducing problem-based learning (PBL) and case-based learning (CBL) into radiation oncology education [17, 18]. It is actually a worldwide issue that the level of medical education in radiation oncology cannot meet the increasing demand. This has been reported by countries such as India [19], Canada [5], Australia [20], and many European countries [2, 4, 21]. Specific problems include fragmented oncology teaching across various anatomical systems, the specialization of oncology making it difficult to teach, and lower understanding of radiotherapy compared to surgery and chemotherapy [2, 20]. Another issue with oncology courses in China is that too little attention is paid to cultivating students’ care for patients compared to countries such as the UK, Australia, and India. The main focus of courses remains on basic concepts, highlighting the need for curriculum reform in the future.

The reasons why radiation oncology education is undervalued among medical students in China are complex. Radiation oncology is classified as a third-level subject in the Chinese education system, indicating its high level of specialization. In contrast to surgery and systemic therapy, which are interdisciplinary with surgical and internal medicine, the knowledge of radiation oncology is relatively specialized and can be difficult for medical students to understand. The development of radiotherapy in China started late, and even today, many hospitals cannot afford radiotherapy equipment or do not have enough medical physicists. This results in students not being exposed to radiotherapy in clinical practice. In most medical colleges in China, the radiotherapy department is not included in routine rotations, leaving most students unfamiliar with the radiotherapy process. Additionally, social awareness of radiation oncology is limited.

To meet the increasing demand for radiation oncologists, curriculum reform is necessary in China. One possible direction is to introduce radiation oncology education to resident physicians and develop a study plan that allows doctors from other departments to rotate through the radiotherapy department. Radiation oncology education can help them in the future participation in multidisciplinary teams (MDTs) and foster their multidisciplinary awareness. For students interested in participating in cancer treatment in the future, short-term rotations can broaden their thinking, as demonstrated by standardized training for North American residents [22]. However, this type of rotation is generally applied to graduated residents undergoing routine rotations and is of little help to graduates who participate in oncology-related research or other jobs. Therefore, it is of great significance to increase the duration of radiation oncology courses, and to include practical courses during the undergraduate period to improve medical students’ oncology literacy.

Since its inception, MOOCs have been widely used in medical education across various fields. Previous cases in areas such as oncology, geriatrics, health care, and others have indicated that MOOC-based teaching can effectively enhance students’ knowledge and demonstrate results comparable to traditional offline teaching [12–14, 23]. In this study, MOOCs were used as a complement to offline teaching, effectively improving students’ knowledge levels and increasing their interest in learning. Moreover, compared to offline teaching, MOOCs offer more flexible scheduling and enable students to review materials conveniently through video replay, facilitating further consolidation of knowledge. Therefore, the application of MOOCs in radiation oncology education is worthy of further consideration and exploration.

This study has several limitations. To ensure baseline consistency, the survey population was restricted to medical students at the clerkship stage. The course only included two demonstration lessons, resulting in an incomplete curriculum structure. Additionally, teacher supervision was not optimal, which may have introduced bias. Due to time constraints, many new technologies in radiation oncology were not introduced, and the lessons focused only on cervical cancer, with other diseases not being discussed in detail. In the future, it is hoped that a complete curriculum design for introducing radiotherapy for various organs and systems can be developed.

## Conclusion

In this study, we implemented a series of MOOCs for practical courses in radiation oncology and evaluated their effectiveness through pre- and post-test questionnaires. Our results indicate that MOOCs can effectively engage students’ interest in radiation oncology and enhance their knowledge of the subject. Given the challenges facing radiation oncology education in China, our study suggests a potential solution. The radiation oncology education in China faced challenges, and the study shows us a possible way out. By integrating online and offline practical courses for undergraduates and offering short-term courses to graduates, we may attract more talents to the field and foster its development through positive feedback.

## Data Availability

The authors confirm that the data supporting the findings of this study are available within the article.
